# Genome-wide mutagenesis and multi-drug resistance in American trypanosomes induced by the front-line drug benznidazole

**DOI:** 10.1038/s41598-017-14986-6

**Published:** 2017-10-31

**Authors:** Mônica C. Campos, Jody Phelan, Amanda F. Francisco, Martin C. Taylor, Michael D. Lewis, Arnab Pain, Taane G. Clark, John M. Kelly

**Affiliations:** 1 Department of Pathogen Molecular Biology, London School of Hygiene and Tropical Medicine, Keppel Street, London, WC1E 7HT UK; 2 Pathogen Genomics Laboratory, BESE Division, King Abdullah University of Science and Technology (KAUST), Thuwal, 23955-6900 Saudi Arabia; 3 Department of Infectious Disease Epidemiology, London School of Hygiene and Tropical Medicine, Keppel Street, London, WC1E 7HT UK

## Abstract

Chagas disease is caused by the protozoan parasite *Trypanosoma cruzi* and affects 5–8 million people in Latin America. Although the nitroheterocyclic compound benznidazole has been the front-line drug for several decades, treatment failures are common. Benznidazole is a pro-drug and is bio-activated within the parasite by the mitochondrial nitroreductase TcNTR-1, leading to the generation of reactive metabolites that have trypanocidal activity. To better assess drug action and resistance, we sequenced the genomes of *T. cruzi* Y strain (35.5 Mb) and three benznidazole-resistant clones derived from a single drug-selected population. This revealed the genome-wide accumulation of mutations in the resistant parasites, in addition to variations in DNA copy-number. We observed mutations in DNA repair genes, linked with increased susceptibility to DNA alkylating and inter-strand cross-linking agents. Stop-codon-generating mutations in *TcNTR-1* were associated with cross-resistance to other nitroheterocyclic drugs. Unexpectedly, the clones were also highly resistant to the ergosterol biosynthesis inhibitor posaconazole, a drug proposed for use against *T. cruzi* infections, in combination with benznidazole. Our findings therefore identify the highly mutagenic activity of benznidazole metabolites in *T. cruzi*, demonstrate that this can result in multi-drug resistance, and indicate that vigilance will be required if benznidazole is used in combination therapy.

## Introduction

Chagas disease results from infection with the insect-transmitted protozoan parasite *Trypanosoma cruzi*. It is a life-long infection and a major cause of morbidity and mortality in many areas of Latin America^1^. Recently, the disease has also become more widespread, with large numbers of infected individuals being diagnosed within migrant populations, particularly in Europe and the USA^2^. In the initial ‘acute’ stage, which occurs 4–6 weeks post-infection, Chagas disease generally manifests as a transient low-level febrile condition. In children however, acute stage infections can lead to myocarditis or meningoencephalitis, and are sometimes fatal. Following suppression of the acute infection by a robust adaptive immune response^3^, the disease proceeds to a long-lasting asymptomatic chronic stage, which is characterised by an extremely low parasitemia. About one-third of those infected will eventually advance to a symptomatic stage, although this can take decades. Most of these individuals will develop cardiomyopathy^4^, or less commonly, digestive tract megasyndromes, outcomes for which there are few therapeutic options.

Although benznidazole has been used against chronic *T. cruzi* infections for almost 50 years, treatment failures are frequently reported, with non-curative outcomes varying between 6% and 50% in recent clinical trials^5,6^. Other problematic factors include the extended treatment length (often 60–90 days) and frequent toxic side-effects. Furthermore, there is a demonstrated risk for cross-resistance with the other front-line drug, nifurtimox. This arises because both of these nitroheterocyclic agents require to be activated by the *T. cruzi* flavin-dependent mitochondrial type 1 nitroreductase, TcNTR-1^7,8^. Our understanding of how benznidazole kills the parasite and why treatment is often non-curative is limited. The extent to which this is due to acquired resistance or patient non-compliance remains to be determined. In addition, *T. cruzi* is an extremely diverse species and there is extensive natural variation in susceptibility to benznidazole^9,10^, both within and between lineages. Despite this, there is as yet no unequivocal evidence for a link between parasite taxonomic designation and treatment outcome in humans^11,12^, and no definitive mechanism demonstrated that accounts for natural variations in susceptibility. This is an area that requires further study.

Evidence suggests that trypanocidal activity could be mediated, at least in part, by DNA damage^13,14^ caused by reactive metabolites that are generated following reductive metabolism of the drug by TcNTR-1^15,16^. Attempts to dissect the mechanisms underlying treatment failure have been compounded by the extremely low and focal nature of the parasite burden during chronic stage disease^17,18^ and technical difficulties in establishing parasitological cure/non-cure^19^. To gain insight into the mechanisms of resistance, we sequenced the genomes of three drug-resistant clones from a single parasite population that had undergone benznidazole-selection *in vitro*. Here, we show that long-term treatment with benznidazole can give rise to genome-wide mutagenesis in *T. cruzi*, and result in phenotypic changes that include the acquisition of multi-drug resistance.

## Results

### Genome-wide mutagenesis in benznidazole-resistant clones

Following the isolation of three drug-resistant clones from a single *T. cruzi* population exposed to benznidazole selection, we identified a stop-codon-generating mutation in the *TcNTR-1* gene^20^. However, this nonsense mutation by itself was insufficient to account for the high level and inter-clonal diversity of benznidazole-resistance (9–26 fold), indicating that additional mechanisms must operate. To investigate this, we sequenced the genomes of the parental *T. cruzi* Y strain and the three benznidazole-resistant clones using an Illumina HiSeq. 2500 platform (x377 coverage). Genome size was 35.54 Mb (48.3% GC content), distributed over 150,022 contigs. Assembly, using the SPAdes program, and annotation performed with the Companion Sanger Pipeline (https://companion.sanger.ac.uk) on the 3,000 largest contigs, identified 8,289 putative protein coding genes, 5119 with predictable function. The *T. cruzi* CL Brener genome strain^21^ was used as reference. The parental Y strain genome sequence and those of the three drug-resistant clones were mapped using the BWA-mem alignment software, and single nucleotide polymorphisms (SNPs) were called using the SAMtools package.

**Figure 1:**
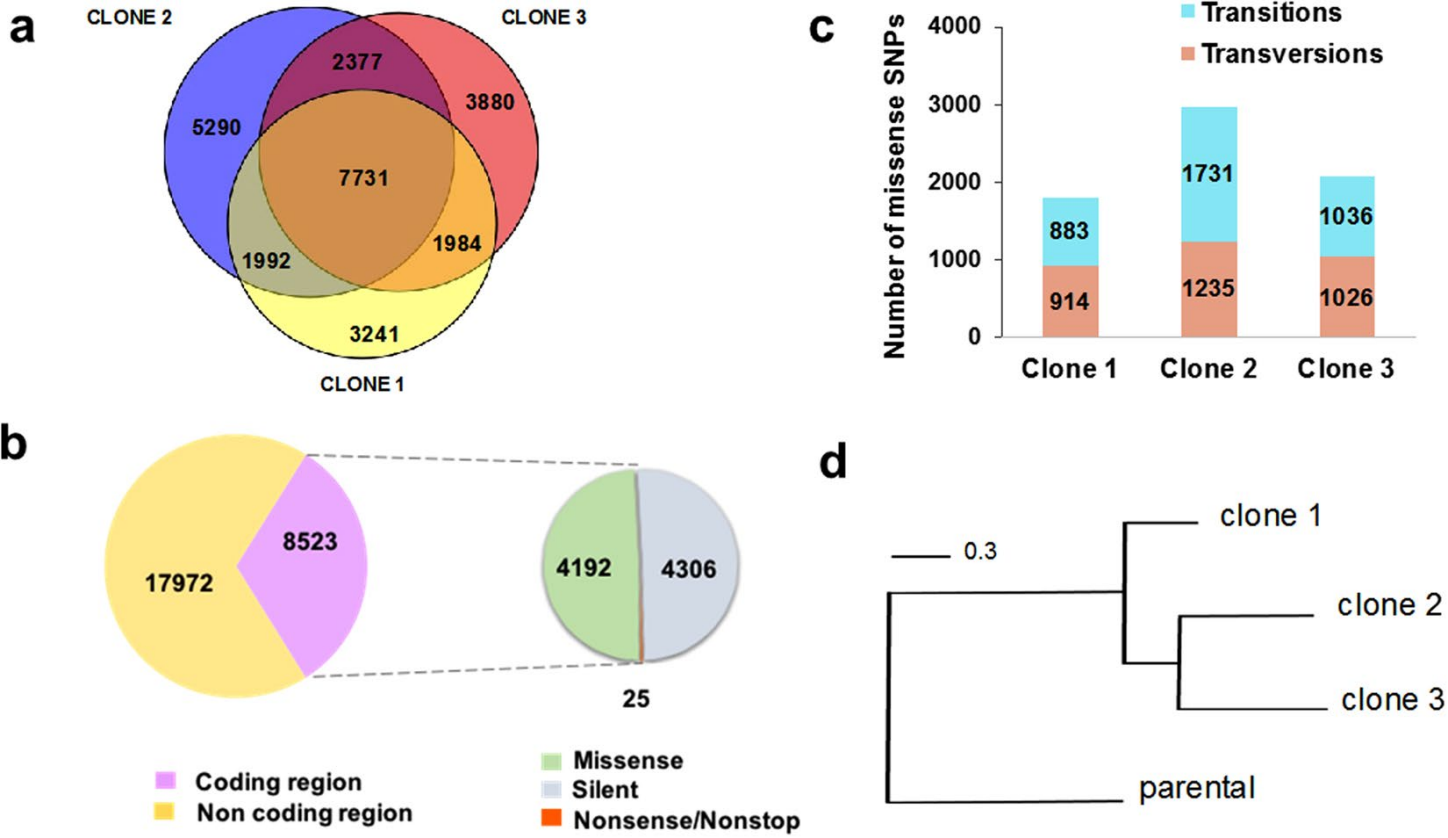
Analysis of SNPs identified in *T. cruzi* clones selected for benznidazole-resistance. (**a**) Venn diagram illustrating the distribution of 26,495 distinct SNPs identified by genome sequencing of three drug-resistant clones. (**b**) The total number of SNPs in coding and non-coding genomic regions are shown in purple and yellow. The smaller circle (right) illustrates SNP distribution within coding regions: missense (green), silent (grey) and nonsense/nonstop (orange). (**c**) Classification of missense SNPs as transitions (purine ↔ purine; blue) or transversions (purine ↔ pyrimidine; pink). (**d**) Maximum-likelihood phylogenetic tree based on nucleotide alignment (FastTree program), showing relationship between parental and drug-resistant *T. cruzi* clones. Scale bar represents the number of substitutions per site.

We found evidence of the genome-wide accumulation of mutations in the drug-resistant clones. There was a total of 26,495 point mutations (Fig. 1a), with 0.42 SNPs/kb in clone 1, 0.49/kb in clone 2, and 0.45/kb in clone 3. 32% of SNPs were in coding regions, with an equivalent number of synonymous and non-synonymous changes (Fig. 1b). A total of 25 nonsense/nonstop mutations were identified (Supplementary Table S1), including the internal stop codon within *TcNTR-1* ^20^. Transitions (A ↔ G or C ↔ T) were significantly more frequent than transversions in clone 2, although in clones 1 and 3 the numbers were similar (Fig. 1c). 7,731 of the SNPs were present in all three clones and phylogenetic analysis suggested that the lineage giving rise to clones 2 and 3 diverged from a common ancestor of clone 1 during the drug-selection process (Fig. 1d). It can be inferred therefore that SNPs common to clones 1 and 2, and to clones 1 and 3 (Fig. 1a), arose from point mutations acquired by the ancestral drug-resistant line and that subsequent differential gene conversion led to their reversion in clones 3 and 2, respectively.

**Figure 2:**
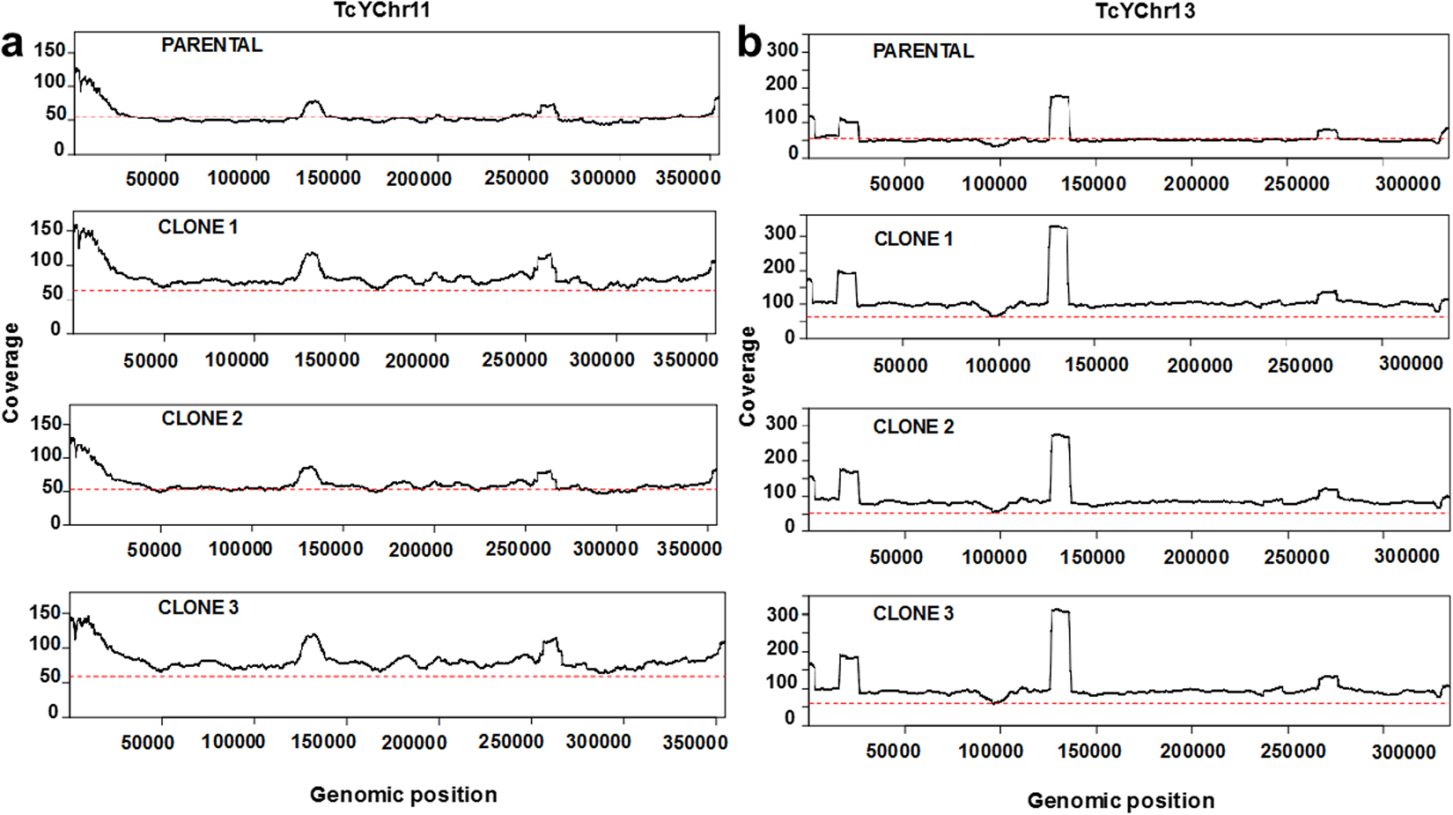
Large-scale DNA copy-number variation in benznidazole-resistant clones. Histograms showing read-coverage for specific regions of chromosome 11 (**a**) and 13 (**b**) in parental and drug-resistant clones. Chromosome annotation was performed by ordering the contigs against the *T. cruzi* CL Brener reference genome^21^. High read peaks correspond to repetitive hypothetical protein genes, tandem repeats within kinesin-like genes (chromosome 11), linked histone H2A genes and short interspersed DNA sequences (chromosome 13). The red dotted line represents the average read-coverage across all chromosomes (single-copy genes). Assessment by the program control-freeC is consistent with an additional copy (trisomy) of the regions shown in chromosome 11 [from TCRUZ.110005800 (TcCLB.509349.30) to TCRUZ.110020000 (TcCLB.506443.80)] and chromosome 13 [from TCRUZ_130005000 (TcCLB.510165.60) to TCRUZ_130019000 (TcCLB.508325.90)]. Chromosome 11 in clone 2 did not display an increased read-coverage compared to the genome-wide average.

Copy number variation (CNV) and read coverage across the data set were also assessed. Trisomy was predicted for a large region of chromosome 13 in all three drug-resistant clones, on the basis of a 1.5-fold higher read-coverage compared to the genome-wide average (Fig. 2b), although the fragmented nature of the reference genome^21^ prevents us from making unequivocal inferences about the entire chromosome. We also found a similarly increased sequence coverage associated with regions (hundreds of kb) in chromosome 11 (clones 1 and 3), a finding supported by a control-freeC analysis. Genome plasticity has been widely observed in *T. cruzi* ^22^. With benznidazole, where resistance can arise by a “loss-of-function” mechanism, parasites missing an entire copy of the chromosome containing the *TcNTR-1* gene have previously been isolated^7^. In this present study, where we observed that resistance was associated with copy number increases in chromosomes 11 and 13, the most parsimonious explanation is that enhanced expression of one or more of the linked genes contributes to the selected phenotype. In clone 2, the data suggest a reversion to disomy in chromosome 11 (Fig. 2b), following divergence from clone 3 (Fig. 1d), although the possibility that the increased copy number was acquired independently by clones 1 and 3 cannot be excluded.

### Deficiencies in DNA repair in drug-resistant parasites

**Figure 3:**
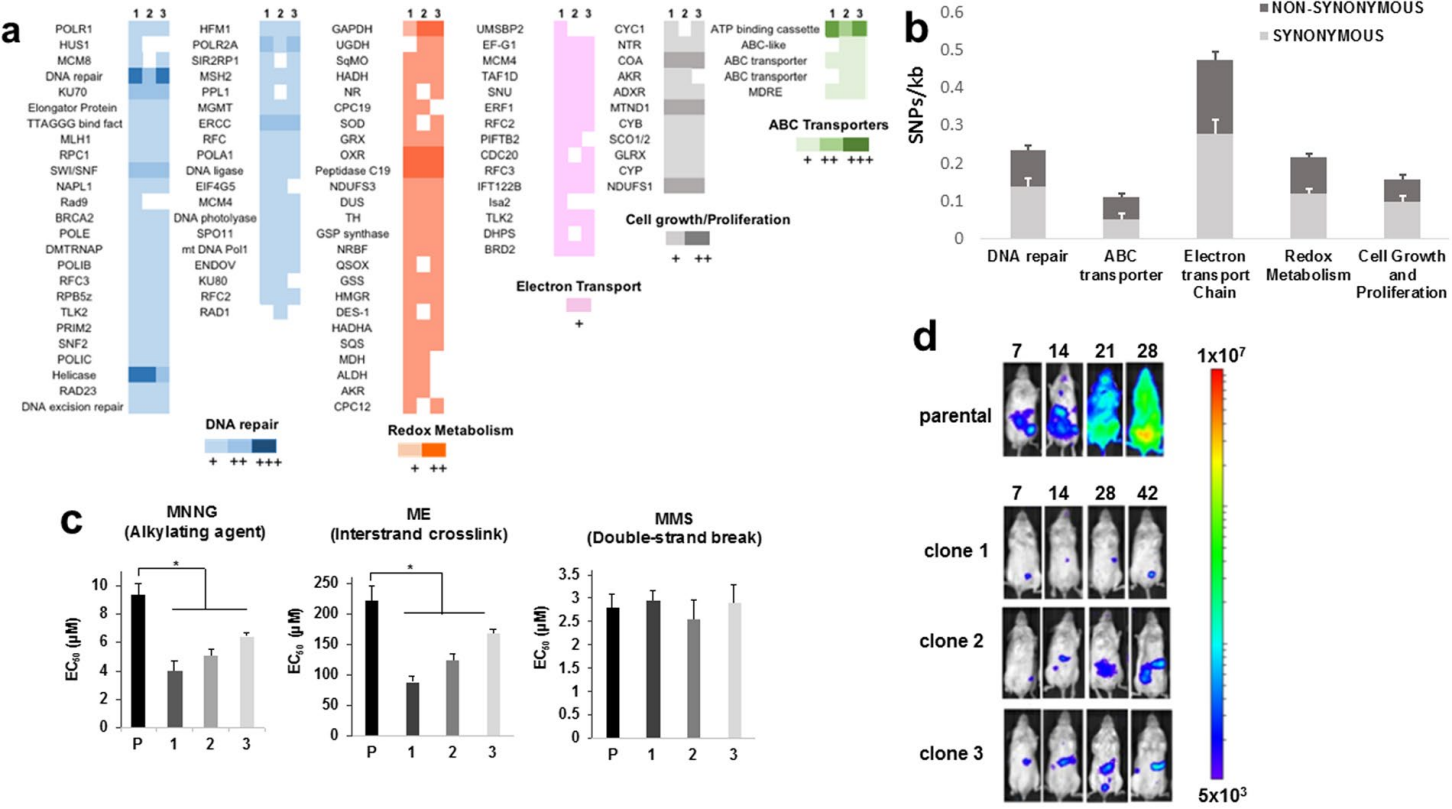
Genetic and phenotypic changes in benznidazole-resistant clones. (**a**) Total point mutations in genes linked to DNA repair, redox metabolism, electron transport, cell growth and proliferation, and ABC transporter activity, in each of the three clones. The colour scale indicates the number of mutations per gene: 1 (+), 2 (++), or 3 or more (+++). (**b**) Average SNPs/kb in genes from different functional categories in the 3 clones. Synonymous and non-synonymous SNPs are shown. (**c**) Sensitivity of parental and drug-resistant parasites to the DNA damaging agents methyl-3-nitro-1-nitrosoguanidine (MNNG), mechlorethamine (ME) and methyl methanesulfonate (MMS) (Methods). Statistical analysis was performed using one-way analysis of variance; *p* values, respectively: 0.02, 0.01, 0.02 (MNNG); 0.002, 0.003, 0.02 (ME). (**d**) Bioluminescence imaging of SCID mice infected i.p. with parental and drug-resistant clones (10^4^ tissue culture trypomastigotes). Parasites were genetically transformed to express the luciferase reporter gene *PpyRe9h* ^17,28^ (Methods). Ventral images of representative mice (n = 3) are shown, with the number of days post-infection given above. Heat maps indicate the intensity of bioluminescence from low (blue) to high (red) (log_10_ scale); the minimum and maximum radiances for the pseudocolour scale are shown. The mice infected with the parental Y strain required euthanization on day 28, in accordance with animal welfare requirements (Methods).

Using gene ontology annotations, it was possible to link mutations to functional categories. The majority occurred in hypervariable regions, including the large and highly diverse surface antigen gene families, such as the mucins and trans-sialidases, which constitute much of the parasite genome^23^. In functional categories or metabolic pathways that have been tentatively associated with drug-resistance^8,14,16,24–27^, there were a range of SNP frequencies ranging from 0.47/kb in genes linked to electron transport to 0.11/kb in ABC transporter genes (Fig. 3a and b). Individually or collectively, it is implicit that changes to the sequence or expression of these, or other genes, have contributed to the enhanced levels of benznidazole-resistance. Given the extensive number of mutations in DNA repair enzymes, we investigated the susceptibility of the drug-resistant clones to DNA damaging agents. This revealed enhanced sensitivity to compounds that promote DNA alkylation (1-methyl-3-nitro-1-nitrosoguanidine) or inter-strand cross-linking (mechlorethamine), but not to methyl methanesulfonate, which causes double-strand breaks (Fig. 3c).

To determine if mutagenesis had compromised the infectivity of the drug-resistant clones, we inoculated immunocompromised SCID mice with parasites transformed to express a red-shifted bioluminescence reporter^17,28^ (Methods). Whereas the non-resistant parental line resulted in a fulminant infection, which required the mice to be euthanised after 28 days, infections with drug-resistant clones were asymptomatic, and there was an extremely low parasite burden (Fig. 3d). This loss of fitness may reflect that infectivity was not a trait under selection during the continuous *in vitro* culturing used to generate benznidazole-resistant parasites (Methods). Studies had previously shown that there were no significant differences in the rate of intracellular amastigote replication or infectivity of cultured mammalian cells^20^.

### Multi-drug resistance following benznidazole selection

**Figure 4:**
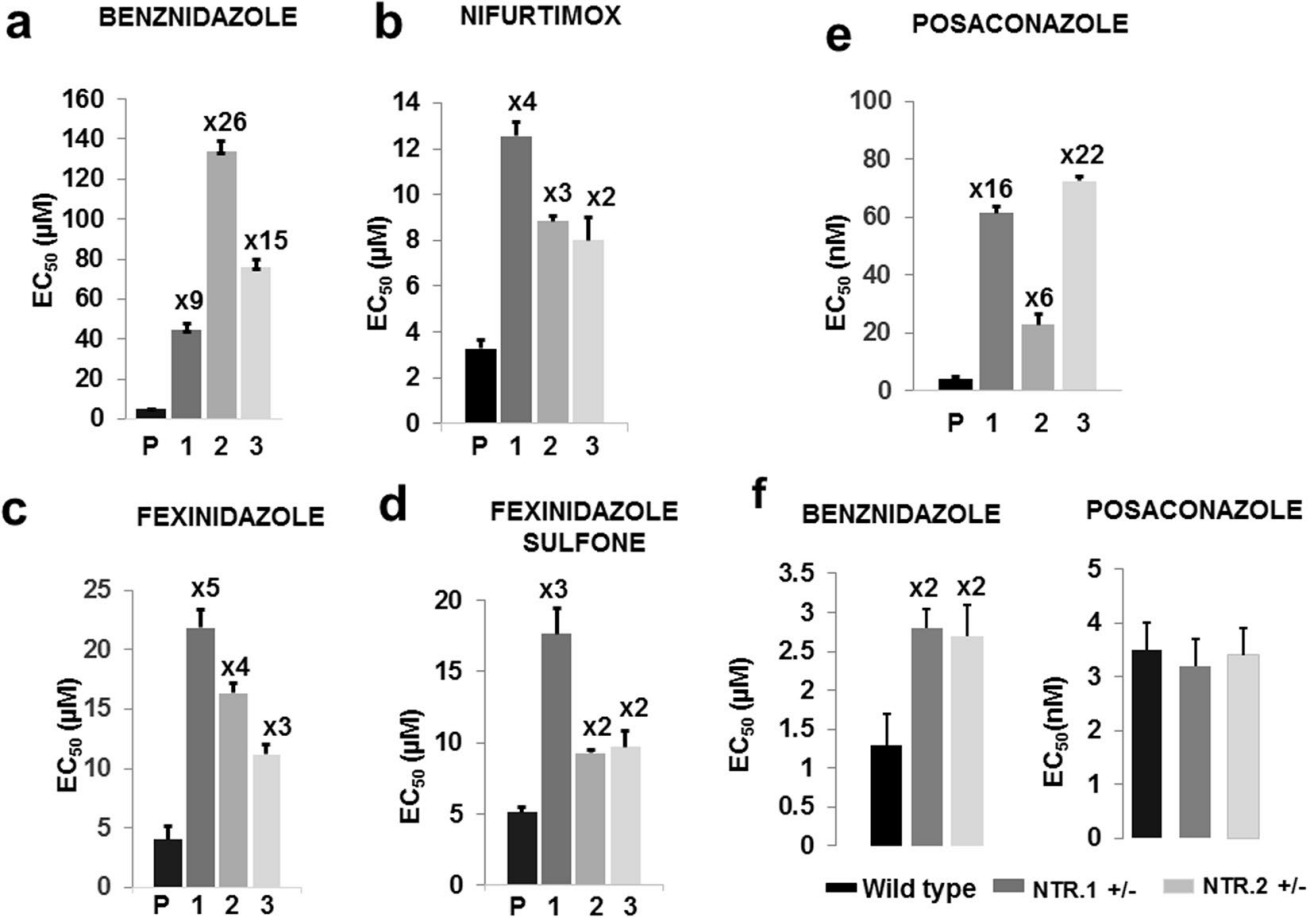
Multi-drug resistance following benznidazole-selection. (**a**–**e**) EC_50_ values were obtained (Methods) for the parental Y strain (P) and drug-resistant clones 1–3 (epimastigotes) against benznidazole, nifurtimox, fexinidazole, fexinidazole sulfone and posaconazole. The fold-resistance is indicated above each bar. (**f**) Benznidazole and posaconazole susceptibility of parental parasites and two genetically modified clones in which one copy of the *TcNTR-1* gene had been deleted. Each experiment was performed 3 times in triplicate.

As predicted for parasites with a disrupted *TcNTR-1* gene, the three clones displayed cross-resistance to other nitroheterocyclic agents (Fig. 4a–d; Supplementary Table S2), including nifurtimox, a drug which is also used to treat *T. cruzi* infections, and fexinidazole, a drug which has undergone clinical trials against Chagas disease. The level of resistance (2–5 fold) was consistent with that previously observed for nitroheterocyclic compounds following disruption of one copy of *TcNTR-1* ^7,29^. It can therefore be inferred that the other, as yet unknown mechanisms that lead to the enhanced benznidazole-resistance, do not contribute significantly to additional levels of cross-resistance against other nitroheterocyclic drugs. Unexpectedly, we found that each of the clones were also highly resistant to the anti-fungal agent posaconazole (6–22 fold, Fig. 4e). Although posaconazole monotherapy recently failed in clinical trial against Chagas disease^5,30^, its use in combination with benznidazole has been discussed^31–33^ and subjected to preliminary trial in humans^30^. Posaconazole is an ergosterol biosynthesis inhibitor that targets lanosterol 14α-demethylase (CYP51)^34,35^, a mechanism of action that shares no known biochemical overlap with that of benznidazole. In the drug-resistant clones, there were no mutations detected in *CYP51*, and the gene is not located on either of the chromosomes (11 and 13) which displayed increased copy number. Mutations were noted in the 5’-upstream region of *CYP51*, and in the coding regions of other genes linked with ergosterol biosynthesis (Supplementary Figure S1). However, these did not affect the intracellular level of CYP51, as judged by binding between the enzyme and posaconazole conjugated with the fluorophore boron-dipyrromethene^36,37^ (Supplementary Figure 2). To exclude the possibility that TcNTR-1 depletion might perturb susceptibility to posaconazole, we tested its activity against two cloned *T. cruzi* cell lines (Methods) in which we had deleted one copy of the gene (Fig. 4f). There was no significant difference compared to the parental parasites.

## Discussion

Investigating the underlying causes of treatment failure in humans has been challenging in the case of *T. cruzi* infections^5,6^. Reasons include the possible influences of host and parasite genetics, the effects of toxicity on compliance, difficulties in demonstrating parasitological cure, the unresolved contribution of drug-resistance and the possibility of re-infection/co-infection. With benznidazole, there are no data available from clinical trials that provide insight into the mechanisms of resistance or the precise mode of drug action in infected individuals. Evidence from *in vitro* studies suggest that the pivotal step in both drug activity and resistance involves bio-activation of the drug by the nitroreductase TcNTR-1^7–9,15,16^. This gives rise to toxic metabolites that react with a range of biological molecules (for review, see ref.^8,29^). However, given the high level of resistance that is achievable *in vitro* (Fig. 4, as example), and the wide range of natural susceptibility to benznidazole^9,10^, it is clear that other mechanisms can also contribute. For example, several other parasite genes have been identified that could act through alternative bioactivation^38^, alleviation of oxidative stress^39^, or increased efflux^26^. In the context of DNA, electrophilic drug metabolites, increased oxidative stress resulting from drug adduct-thiol conjugation, and the generation of oxidised nucleotides such as 8-oxo-guanine, have been implicated as potential mediators of mutagenesis in benznidazole-treated *T. cruzi* ^14–16,40,41^.

Here, we show that benznidazole-induced mutagenic effects can be extensive and genome-wide in *T. cruzi*, with a range of phenotypic consequences, including reduced DNA repair capacity and multi-drug resistance. These types of outcome have many parallels with the situation in drug-resistant cancer, where selective pressure, coupled with the early acquisition of defects in the DNA damage repair machinery, can drive the development of multi-drug resistance through mechanisms linked with chromosome instability and an increased propensity to acquire mutations^42,43^. In a similar way, our results suggest that in *T. cruzi* under benznidazole-selection, the combined effects of electrophilic drug metabolites and deficiencies in DNA repair, which could arise early in the process, might also act in concert to create a hypermutagenic environment that can drive the development of enhanced resistance and other phenotypic changes (Fig. 3d). Reduced infectivity was one was one of the acquired traits common to the drug-resistant clones. This phenotype could have arisen as a result of sequence changes at a single locus, or could represent the collective effect of multiple mutations. It will be important to determine whether this loss of fitness is a frequent or inevitable consequence of benznidazole-resistance, or whether selective pressures encountered in an *in vivo* context would be sufficient to exclude this particular outcome.

The current benznidazole dosing regimen involves daily administration for periods of 60 days or more, with many patients failing to complete treatment because of toxic side-effects^5,6,30^. Partly to avoid this, combination therapy with posaconazole has been suggested as a treatment option that might allow a reduction in patient exposure to benznidazole^30^. On the basis that benznidazole and posaconazole activities are not thought to overlap, it has also been assumed that the generation of cross-resistance using this regimen would be unlikely. However, our results show that this need not be the case. Parasites selected on benznidazole alone were found to have developed high-level resistance to posaconazole (Fig. 4e). This outcome could not be attributed to changes in the *CYP51* gene, and presumably results from the effects of other mutation(s), either individually or collectively. Whatever the precise mechanism, these findings have implications for future Chagas disease strategies, suggesting that combination therapy involving benznidazole will require careful monitoring, irrespective of the secondary drug used. Although caution must be displayed in extrapolating findings made *in vitro*, our data further highlight an urgent need for studies aimed at isolating parasites from patients following non-curative benznidazole therapy, to determine if induced mutagenesis and acquired drug-resistance have contributed to treatment failure. However, there will be significant technical and analytical issues associated with this. As mentioned previously^44^, these include complexity resulting from multi-clonal infections, possible stage-specific differences in parasite susceptibility, the tissue origin of recrudescence, interplay with the immune system, and finally, difficulties in isolating parasites from chronic chagasic patients, both before and after treatment.

## Methods

### Isolation of benznidazole-resistant clones

*T. cruzi* (Y strain) was cultured as epimastigotes in supplemented RPMI-1640 medium at 28 °C^45^. Parasites were selected for benznidazole-resistance by culturing in the continuous presence of 182 μM benznidazole for 10 passages. From the 11^th^ until the 15^th^ passage, the drug concentration was increased in 10 μM steps^46^. The total selection period was 4 months. Parasites were then cloned by limiting dilution^20^ and the extent of benznidazole-resistance assessed. The resistance phenotype was found to be unchanged after 6 months continuous culturing in the absence of benznidazole^46^.

### Genome sequencing

Genomic DNA was prepared from three drug-resistant clones and the parental strain, and then submitted to paired-end sequencing (100 bp) using an Illumina HiSeq. 2500 platform. Assembly of high-quality reads was conducted using SPAdes assembly program, with genome annotation on the 3,000 largest contigs performed by the Companion Sanger Pipeline (https://companion.sanger.ac.uk), using the *T. cruzi* CL Brener strain^21^ as reference. Mapping of the parental population to the three drug-resistant clones and SNP analysis were performed using the BWA-mem alignment software and SAMtools package, respectively. Gene functional categories were classified based on GO annotations. Copy number variation was assessed using the program control-freeC (http://bioinfo-out.curie.fr/projects/freec/tutorial.html). Sequence data from this paper are available on the website PathogenSeq LSHTM (http://pathogenseq.lshtm.ac.uk/t_cruzi/) and at the European Nucleotide Archive (http://www.ebi.ac.uk/ena/) under the accession no. PRJEB14637; clone 1 (ERS1913021), clone 2 (ERS1913022), clone 3 (ERS1913023), parental Y strain (ERS1913024).

### Genetic modification of *T. cruzi* and assessment of infectivity by *in vivo* imaging

The red-shifted luciferase gene *PpyRE9h* was integrated into rRNA loci in three drug-resistant clones and the parental Y strain using the construct pTRIX2-RE9h^17^ (Fig. 3d). Clones expressing comparable levels of bioluminescence were cultured under conditions that promote differentiation into metacyclic trypomastigotes^17^. The resulting parasites were used to infect rat myoblast L6 monolayers at a ratio of 10 metacyclics per host cell. Following incubation at 37 °C under 5% CO_2_ for 7 days, tissue culture trypomastigotes were collected from the supernatant and female CB17 SCID mice (aged 8–12 weeks and bred in-house) were infected i.p. with 10^4^ trypomastigotes. Animals were maintained under specific pathogen-free conditions in individually ventilated cages, and experienced a 12 hour light/dark cycle, with access to food and water *ad libitum*. To monitor infections, mice were injected with 150 mg kg^−1^ d-luciferin every 7 days and imaged using an IVIS Lumina II system (Caliper Life Science), as described previously^17^. Mice injected with the parental line developed fulminant infections and were euthanized at or before humane end-points by exsanguination under terminal anaesthesia. All animal work was performed under UK Home Office licence 70/8207 and approved by the London School of Hygiene and Tropical Medicine Animal Welfare and Ethical Review Board.

To generate parasites in which one copy of the *Tc-NTR-1* gene had been disrupted, bioluminescent *T. cruzi* CL Brener epimastigotes^17,47^ were transfected as described previously^7^. Briefly, parasites were electroporated with plasmid pKO-NTR-BLA which had been linearised by *Kpn* I/*Sac* II digestion, and blasticidin-resistant parasites were isolated, cloned and checked by PCR to confirm integration and disruption.

### Parasite susceptibility to drugs or DNA damaging agents

Epimastigotes were seeded at 2.5 × 10^5^ ml^−1^ in 96-well plates in the presence of a range of concentrations of nitroheterocyclic compounds (benznidazole, nifurtimox, fexinidazole, or fexinidazole sulfone), posaconazole, or DNA damaging agents (MNNG, ME and MMS). Plates were incubated for 4 days at 28 °C, with resazurin added for the final 48 hours. The plates were then read in a Spectramax plate reader and the EC_50_ values determined using GraphPad Prism. Statistical analysis was performed using one-way analysis of variance, with significance for *p* values < 0.05 (*).

## Electronic supplementary material

Supplementary data
