# Supplementary material for: Genome-wide mutagenesis and multi-drug resistance in American trypanosomes induced by the front-line drug benznidazole
Source: Sci Rep. 2017 Oct 31;7:14407. doi: 10.1038/s41598-017-14986-6 (PMC5663738; doi:10.1038/s41598-017-14986-6)
Supplement: Supplementary file 1 — Supplementary data [file 41598_2017_14986_MOESM1_ESM.pdf]

Genome-wide mutagenesis and multi-drug resistance in American trypanosomes induced by the front-line drug benznidazole

Mônica C. Campos,[1] Jody Phelan,[1] Amanda F. Francisco,[1] Martin C. Taylor,[1] Michael D. Lewis,[1] Arnab Pain,[2] Taane G. Clark[1,3] and John M. Kelly[1*]

Supplementary data: Tables S1 and S2; Figures S1 and S2

Supplementary Figure 1. Mutations associated with ergosterol biosynthesis in drug-resistant *Trypanosoma cruzi* clones. (**a**) An overview of the biosynthetic pathway highlighting genes that have acquired mutations (red), and identifying the reaction catalysed by lanosterol 14α-demethylase (CYP51). (**b**) Missense mutations in genes involved in *T. cruzi* ergosterol biosynthesis. (**c**) Mutations in the region immediately upstream of the *CYP51* gene.

Supplementary Figure 2. Analysis of intra-parasite posaconazole-CYP51 interaction using flow cytometry. *T. cruzi* epimastigotes ($1x10^6$) (parental Y strain and the three benznidazole-resistant clones) were incubated for 5 minutes with 2 µM posaconazole conjugated with the fluorophore boron-dipyrromethene (POS-BODIPY). Binding of POS-BODIPY to CYP51 disrupts the stacking interaction between the fluorophore moiety and the aromatic rings of the drug, rendering the compound highly fluorescent. For the assay, 10,000 events were analyzed and the epimastigotes selectively gated. Data are expressed as the mean of BODIPY fluorescence (emission λ= 506nm) in each parasite set: parental Y strain not treated (grey), and parental (blue), clone 1 (red), clone 2 (orange) and clone 3 (green) parasites treated with POS-BODIPY. The WinMDI software package was used for data analysis. There were no significant differences between parental parasites and the drug-resistant clones and no association with the extent of posaconazole resistance.

a

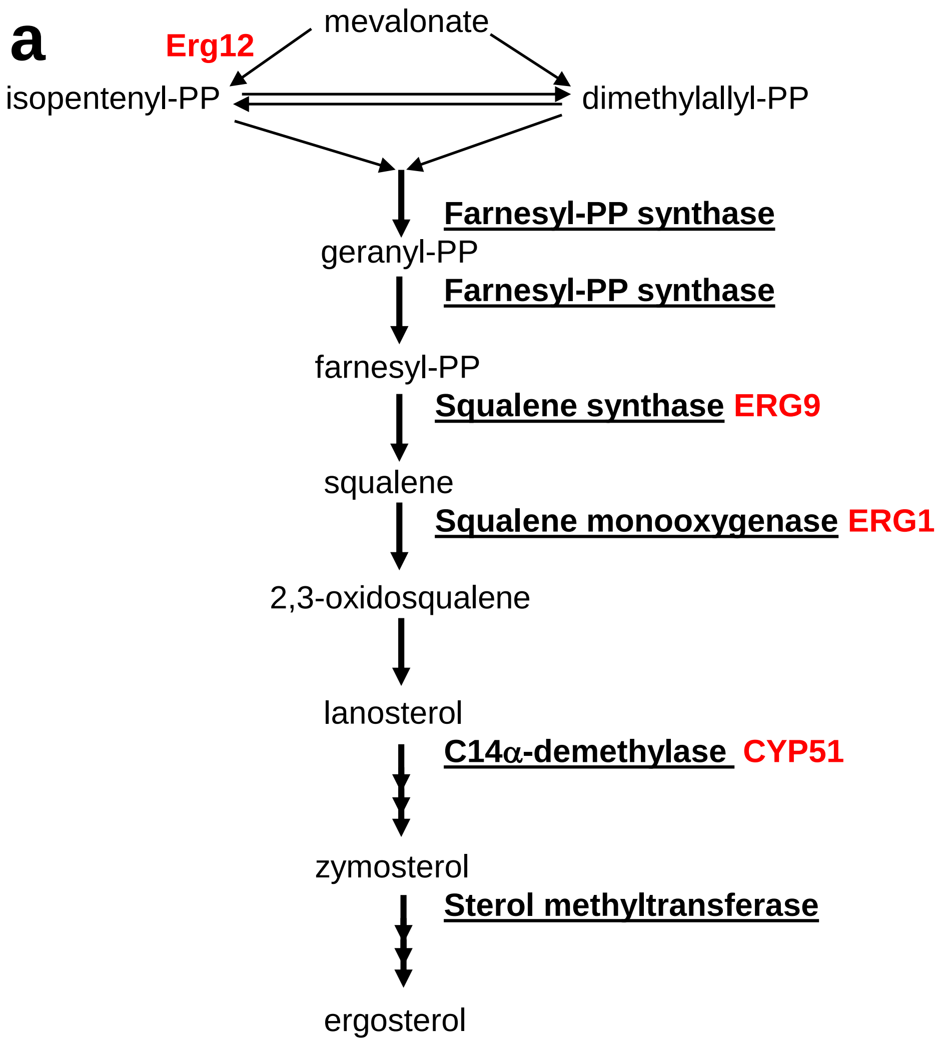

b

Gene name	Chromosome	Position	Nucleotide changes (missense)		
			Clone 1	Clone 2	Clone 3
Erg 12 (mevalonate kinase)	TCRUZ_31	391046		G->T	G->T
Erg 1 (squalene monooxygenase)	TCRUZ_14	414731	T->G	T->G	T->G
Erg 9 (squalene synthase)	TCRUZ_31	66182	A->C	A->C	A->C

c

Gene Name	Chromosome	Position	Nucleotide changes (upstream gene variant)		
			Clone 1	Clone 2	Clone 3
CYP51	TCRUZ_27	70702	C -> T		C -> T
CYP51	TCRUZ_27	71525	C -> T		
CYP51	TCRUZ_27	71532		G -> T	G -> T
CYP51	TCRUZ_27	71563		C -> T	
CYP51	TCRUZ_27	71565		C -> T	

Supplementary Figure 1

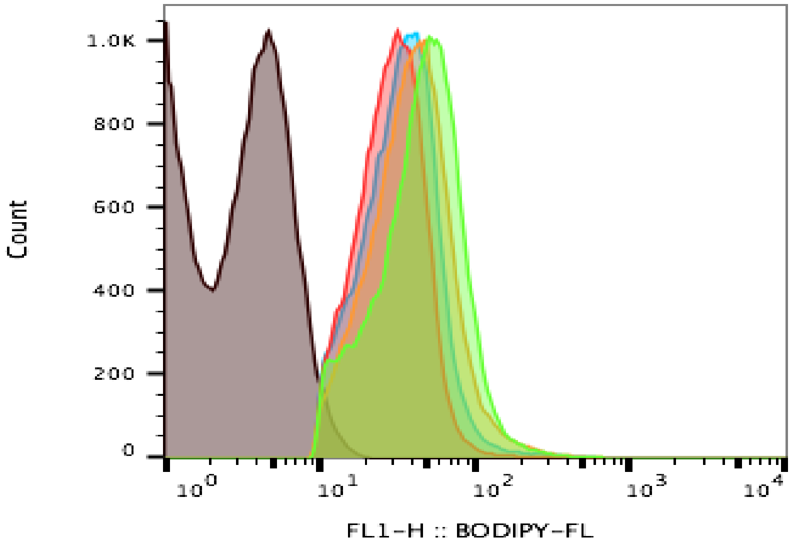

Cell line	fluorescence
Parental – not treated	2.4
Parental – treated	40.2
Clone 1	34.0
Clone 2	47.9
Clone 3	53.9

Supplementary Figure 2

Supplementary Table 1. Nonsense and nonstop mutations detected in *T. cruzi* clones selected for benznidazole-resistance

Gain of stop codon

Gene Name	Chromosome	Position	Nucleotide changes		
			Clone 1	Clone 2	Clone 3
Concanavalin A-like lectin/glucanases superfamily	TCRUZ_00	1087576		C → G	
BNR repeat-like domain containing protein	TCRUZ_2	87197		G → T	
Elongation factor 1 gamma	TCRUZ_14	158587	G → A	G → A	G → A
NADH-cytochrome b5 reductase	TCRUZ_14	260955	C → T	C → T	C → T
Hypothetical protein	TCRUZ_18	122195	G → T	G → T	G → T
Hypothetical protein	TCRUZ_28	2033	A → G		A → G
Nitroreductase	TCRUZ_28	53287	C → T	C → T	C → T
Aminopeptidase	TCRUZ_31	195692		C → A	
Adenylate and Guanylate cyclase catalytic domain containing protein	TCRUZ_35	453172		G → A	
Hypothetical protein	TCRUZ_36	189116			G → T
Glucose-6-phosphate dehydrogenase	TCRUZ_36	1026428	C → G		
Hypothetical protein	TCRUZ_39	1447230			G → A
Hypothetical protein	TCRUZ_40	532683	C → T		
Phospholipid:diacylglycerol acyltransferase, putative	TCRUZ_41	383509	A → T	A → T	A → T

Loss of stop codon

Gene Name	Chromosome	Position	Clone 1	Clone 2	Clone 3
Hypothetical protein	TCRUZ_00	176037	T → G		T → G
Hypothetical protein	TCRUZ_00	1181457		A → C	
Retrotransposon hot spot protein	TCRUZ_14	80072		G → T	G → T
Flagellar radial spoke component	TCRUZ_14	87763	T → A		T → A
Neutral sphingomyelinase activation associated factor-like protein	TCRUZ_22	403964	A → C	A → C	A → C
Fucose kinase	TCRUZ_25	305903	T → G	T → G	T → G
Hypothetical protein	TCRUZ_26	462012		G → C	
Hypothetical protein	TCRUZ_29	98582	A → G	A → G	A → G
CRAL/TRIO domain containing protein	TCRUZ_31	115283	A → G		
5-oxoprolinase	TCRUZ_40	741377	G → T		
Hypothetical protein	TCRUZ_41	206855		A → C	

EC$_{50}$ (6 days)					
	**BNZ (µM)**	**NIF (µM)**	**FEX (µM)**	**FEX-SUL (µM)**	**POS (nM)**
**Parental**	5.1 ± 0.3	3.3 ± 0.4	4.0 ± 1.2	5.2 ± 0.4	3.5 ± 0.5
**Clone 1**	44.5 ± 2.8	12.6 ± 0.6	21.8 ± 1.5	17.6 ± 1.8	55.9 ± 6.5
**Clone 2**	133.7 ± 5.5	8.9 ± 0.2	16.3 ± 0.7	9.3 ± 0.3	21.7 ± 2.4
**Clone 3**	75.6 ± 4.1	8.0 ± 0.5	11.2 ± 1.8	9.7 ± 1.1	78.3 ± 7.1

Supplementary Table 2. Efficacy of nitroheterocyclic compounds and posaconazole against benznidazole-resistant *T. cruzi* clones. *T. cruzi* epimastigotes (parental and drug-resistant clones) were seeded in 96-well plates in the presence of a range of concentrations of benznidazole (BNZ), nifurtimox (NF), fexinidazole (FEX), fexinidazole sulfone (FEX-SUL), or posaconazole (POS) and incubated for 4 days at 28°C, with resazurin added for the final 48 hours (Methods). Statistical analysis was performed using one-way analysis of variance, with significance for p values <0.05 (*).
